# Natural medicines for asthma management: mechanisms aligned with endotypes and multi-dimensional intervention

**DOI:** 10.3389/fphar.2026.1903220

**Published:** 2026-09-03

**Authors:** Jiaxiang Sun, Hui Wang, Xue Li, Ping Wang, Ruibing Li, Sihui Xing, Dandan Wang, Liping Sun

**Affiliations:** 1 School of Traditional Chinese Medicine, Changchun University of Chinese Medicine, Changchun, China; 2 Research Center of Traditional Chinese Medicine, Affiliated Hospital of Changchun University of Chinese Medicine, Changchun, China; 3 Children's Medical Center, Affiliated Hospital of Changchun University of Chinese Medicine, Changchun, China

**Keywords:** airway remodeling, asthma, botanical extracts, inflammation, natural medicines, oxidative stress

## Abstract

**Background:**

Asthma is a heterogeneous chronic inflammatory airway disease affecting over 300 million people globally. Inhaled corticosteroids remain first-line therapy but cause adverse effects and fail to address core pathologies in 5%–10% of patients—particularly those with Type 2-low endotypes exhibiting glucocorticoid resistance. Natural medicines, with multi-component synergy, offer a promising strategy to bridge this gap.

**Objective:**

This review synthesizes advances in botanical extracts and bioactive constituents for asthma management, focusing on pharmacological mechanisms aligned with asthma endotypes.

**Methods:**

A literature search across PubMed and Web of Science (2010–2026) included preclinical studies and randomized controlled trials (RCTs). Therapeutic effects were categorized into anti-inflammation, antioxidant modulation, immunoregulation, and anti-remodeling.

**Results:**

Botanical therapeutics concurrently restore epithelial barrier integrity, rebalance Th1/Th2/Th17 immunity, activate Nrf2-mediated antioxidant pathways, and inhibit airway remodeling. RCTs demonstrate adjunctive benefits in mild-to-moderate asthma; however, translational bottlenecks persist, including phytochemical bioavailability issues and insufficient biomarker-stratified data.

**Conclusion:**

Natural medicines overcome limitations of single-target synthetic drugs via multi-dimensional intervention. Clinical translation necessitates standardized formulations, advanced pulmonary delivery systems, rigorous safety evaluations—including herb-drug interactions—and endotype-stratified RCTs.

## 1 Introduction

Asthma is a heterogeneous chronic airway inflammatory disease affecting over 300 million people worldwide (Jayasooriya et al., 2025; Kim et al., 2025). Epidemiological data from the 2021 Global Burden of Disease Study show a global prevalence of 4,313.76 per 100,000 among children and adolescents ≤20 years old, with the rate in mainland China rising from 0.69% in 1984 to 5.30% in 2021 (Yu et al., 2024a; Li et al., 2025; Wang et al., 2025). Despite a recent downward trend in pediatric prevalence, the annual hospitalization cost for asthma in U.S. children aged 5–17 reaches $260 million, underscoring the persistent health and economic burden of the disease (Barry et al., 2025).

Current management prioritizes long-term, standardized inhaled corticosteroids (ICS) and β_2_-agonists, which effectively suppress airway inflammation in most patients. However, long-term glucocorticoid use is associated with significant adverse effects, including metabolic syndrome, infection susceptibility, hypothalamic-pituitary-adrenal axis inhibition, growth suppression, and osteoporosis (Castillo et al., 2017; Silverberg et al., 2016). Critically, 5%–10% of patients—particularly those with Type 2-low endotypes—exhibit glucocorticoid resistance, characterized by persistent inflammation and poor symptom control despite moderate-to-high dose therapy (Hammad and Lambrecht, 2021; Loke et al., 2002). Leukotriene receptor antagonists (LTRAs), as alternative maintenance therapies, carry risks of neuropsychological adverse events, further limiting treatment options (Tunca et al., 2025). Notably, conventional therapies primarily target immune pathway suppression but fail to address core pathological deficits including epithelial barrier dysfunction, oxidative/antioxidant imbalance, and irreversible airway remodeling—creating a pronounced therapeutic gap for refractory asthma subgroups.

Botanical drugs, characterized by multi-component synergy and multi-target regulatory capacity, offer a promising strategy to bridge this gap. Unlike single-target synthetic agents, plant-derived metabolites inherently modulate interconnected pathological nodes: they concurrently attenuate inflammation, restore epithelial barrier integrity, rectify oxidative stress, and inhibit structural remodeling (Zhou et al., 2022). This multi-dimensional intervention profile aligns precisely with the complex, multi-hit pathogenesis of asthma, positioning botanical agents as ideal candidates for adjunctive or alternative therapies, particularly for patients unresponsive to conventional regimens. This review synthesizes current evidence on botanical drugs for asthma within the framework of modern immune endotype classification. We systematically summarize the pharmacological mechanisms of plant extracts and bioactive metabolites across four core domains: anti-inflammation, antioxidant modulation, immunoregulation, and anti-remodeling. Critically, we move beyond descriptive summaries to provide a critical appraisal of translational bottlenecks—including superficial mechanistic exploration, the disconnect between traditional complex formulations and modern single-compound research, and quality control challenges related to batch variability. Our goal is to provide a rational roadmap for the standardized development and clinical application of botanical anti-asthma agents, supporting the translation of traditional knowledge into evidence-based practice.

## 2 Immunological endotypes and molecular drivers of asthma

Although asthma is defined as a chronic inflammatory airway disease with variable airflow limitation, its clinical heterogeneity suggests it is a syndrome cluster rather than a single disease (Pakkasela et al., 2020; Khalid and Holguin, 2018; Arimura et al., 2025). The same structural changes can be driven by distinct immune axes: the IgE-eosinophil-IL-13-dominated Type 2 cascade or alternative pathways involving Th17/IFN-γ, innate immunity, and metabolic-epithelial dysfunction. This mechanistic divergence explains why some patients fail to respond to inhaled corticosteroids, requiring biologics or surgery. To move beyond phenotypic description, classification has shifted to immune endotypes based on core pathogenic pathways: Type 2-high and Type 2-low (Hammad and Lambrecht, 2021; Ji and Li, 2023). Type 2-high is characterized by Th2/ILC2 activation and T2 cytokines, manifesting as eosinophilia, mucus hypersecretion, and IgE production (Gandhi et al., 2016). Conversely, Type 2-low is often associated with obesity, neutrophilia, and steroid resistance, driven by the Th1/Th17-ILC3 axis and non-T2 cytokines, accompanied by epithelial dysfunction and remodeling (Hammad and Lambrecht, 2021; Liu et al., 2024). Tregs/Th17 imbalance correlates with disease severity, with elevated IL-17 promoting neutrophilic inflammation and steroid resistance in severe cases (Quan-San et al., 2019; Xie et al., 2022; Finotto, 2019). Th1-derived TNF-α and IFN-γ further exacerbate neutrophilic inflammation (Raundhal et al., 2015). Notably, inflammatory pathways often overlap; 73.5% of patients exhibit phenotypic overlap, with 47.5% showing coexistence of Type 2 and non-Type 2 inflammation, complicating precise therapy (Han et al., 2021; Lommatzsch et al., 2006).

**FIGURE 1:**
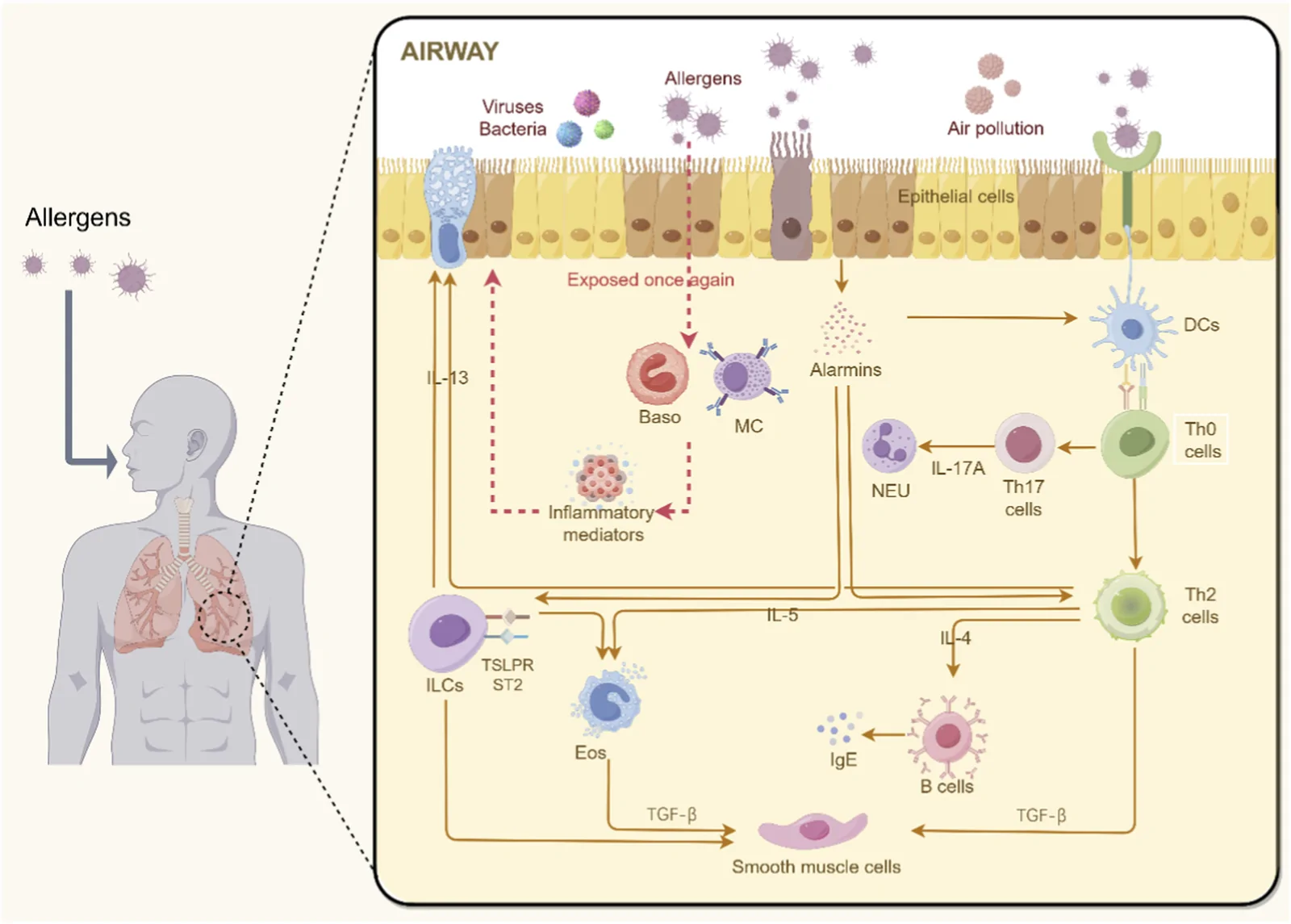
Cellular and molecular interactions initiating innate and adaptive immune responses following stimulation of airway epithelial cells by allergens, pathogens, and air pollutants. Epithelial cells release alarmins that activate DCs, driving the differentiation of Th0 into Th2 and Th17 subsets. Th2 cells secrete IL-4/5/13 to promote IgE production by B cells and Eos activation, while Th17 cells secrete IL-17A to recruit NEU. ILC2, Baso and MC release inflammatory mediators upon stimulation by IL-33 and other signals. TGF-β contributes to the regulation of smooth muscle cell responses, collectively mediating airway inflammation and the immune cascade triggered by repeated allergen exposure.

Type 2-high asthma is the predominant endotype in precision phenotyping, with allergic asthma as its most representative subtype Its pathology conventionally divides into sensitization and effector phases. During sensitization, environmental triggers disrupt epithelial tight junctions, increasing barrier permeability (Roan et al., 2019; Komlósi et al., 2022). Damaged epithelia release alarmins (TSLP, IL-33, IL-25), which activate ILC2s via TSLPR/ST2 receptors and mature dendritic cells (DCs) (Howell et al., 2023). ILC2s rapidly produce IL-5/IL-13, while DCs migrate to lymph nodes to drive Th2 differentiation. Th2 cells secrete IL-4, IL-5, and IL-13 (Hammad and Lambrecht, 2021; Roan et al., 2019; Boonpiyathad et al., 2019). In the effector phase, allergen re-exposure triggers IgE-FcεRI crosslinking on mast cells/basophils, causing degranulation and release of histamine, leukotrienes, and prostaglandins, leading to acute bronchoconstriction and mucus hypersecretion. Chronically, persistent Th2/ILC2/EOS activation drives airway remodeling via TGF-β release, culminating in irreversible airflow limitation (Zhou et al., 2022; Komlósi et al., 2022). Details are shown in Figure 1.

However, not all Type 2-high asthma is allergic. Non-allergic asthma constitutes a heterogeneous spectrum rather than a single phenotype (Handoyo and Rosenwasser, 2009; Chin-See-Chong et al., 2025). While some patients retain Type 2-high features without specific IgE, triggers shift from allergens to viruses, pollutants, or fungi (Chau-Etchepare et al., 2019). Conversely, a refractory subset aligns with the Type 2-low endotype, characterized by neutrophilic/mixed inflammation, innate immune dominance, and links to obesity, smoking, or infections, often resulting in steroid resistance and lung function decline (Hammad and Lambrecht, 2021; Radermecker et al., 2018). Thus, non-allergic asthma is not merely the inverse of allergic asthma; its phenotypes and endotypes exhibit extensive overlap and threshold effects, posing major challenges to precision classification and therapy.

## 3 Downstream pathological consequences and pharmacological targets

While the Type 2-high and Type 2-low endotypes dictate the distinct immunological origins and cytokine landscapes of asthma, they ultimately converge on a shared continuum of tissue-level destructive events. These downstream pathological consequences—namely airway barrier disruption, intracellular inflammatory signal amplification, oxidative stress cascades, and irreversible airway remodeling—not only execute the asthmatic symptoms but also serve as the precise therapeutic anchors for natural multi-target interventions.

### 3.1 Airway barrier damage

The airway epithelial barrier, formed by tight junctions (TJs) comprising the ZO family, occludin, claudins, and adhesion molecules, is central to asthma pathology (Furuse, 2010; Schleimer and Berdnikovs, 2017; Heijink et al., 2020; Roche et al., 1993). Airway epithelial cells (ECs) have shifted from passive defenders to active participants, releasing alarmins upon environmental stimulation to activate Th2/ILC2 responses (Roan et al., 2019; Russell et al., 2024; Tan et al., 2019). Allergens directly degrade TJ proteins, increasing permeability and facilitating sensitization (Wan et al., 1999; Kale et al., 2017). while infiltrating T cells and eosinophils exacerbate barrier dysfunction via cytokine-induced apoptosis and TJ disruption (Tan et al., 2019; Sugita et al., 2018; Trautmann et al., 2002). Natural phytochemicals mitigate this damage by enhancing TJ protein expression/localization, suppressing alarmin hypersecretion, and modulating oxidative stress. By targeting epithelial vulnerability at multiple levels, plant-derived bioactive ingredients provide a novel strategy for preventing and adjunctively treating Type 2-high asthma, addressing root causes beyond symptomatic relief.

### 3.2 Immune imbalance and the cascade of inflammatory responses

Asthma is a complex pathophysiology driven by aberrant activation of multiple immune pathways, traditionally rooted in the Th1/Th2 imbalance characterized by immune cell infiltration and inflammatory factor hypersecretion (Howell et al., 2018). Key cytokines activate the JAK/STAT pathway, exemplified by IL-4–IL-4R–JAK1/2–STAT6 signaling, which transcriptionally regulates inflammation genes (Howell et al., 2018; Mccormick and Heller, 2015; Jasemi et al., 2024). Concurrently, the NF-κB pathway—a central inflammatory regulator—and the MAPK-NF-κB axis amplify asthmatic responses via IL-6/TNF-α production (Alharbi et al., 2021). Toll-like receptors (TLRs), particularly TLR4 in epithelial cells, recognize DAMPs/PAMPs to activate AP-1, NF-κB, and IRF3, playing a pivotal role in house dust mite HDM-induced type 2 inflammation (Aderem and Ulevitch, 2000; Hammad et al., 2009). Given their role in sustaining the asthmatic inflammatory microenvironment, these signaling hubs represent prime therapeutic targets.

### 3.3 Oxidative stress

A hallmark of asthma is the imbalance between excessive reactive oxygen species (ROS) production and deficient antioxidant defenses, exacerbating airway inflammation, barrier dysfunction, and hyperresponsiveness. Oxidative stress and inflammation engage in a vicious cycle: epithelial cells and neutrophils release pro-inflammatory factors that activate NF-κB, upregulate COX-2, and further amplify ROS generation. Counteracting this, Nrf2 serves as the master regulator of antioxidant responses, orchestrating the expression of protective enzymes to mitigate ROS-mediated lung injury (Michaeloudes et al., 2022; Rosanna and Salvatore, 2012). Restoring this impaired Nrf2-driven antioxidant axis represents a compelling therapeutic strategy to break the self-perpetuating cycle of oxidative stress and inflammation in asthma.

### 3.4 Mucus hypersecretion and airway remodeling

Asthma airway mucus hypersecretion arises from the convergence of oxidative stress, inflammation, and epithelial damage (Tan et al., 2019; Corren, 2013). Chronically, these insults drive airway remodeling—a structural substrate for irreversible lung function decline—via epithelial-mesenchymal transition (EMT), fibroblast-myofibroblast transition (FMT), and airway smooth muscle (ASM) proliferation (Heijink et al., 2020; Russell et al., 2024; Tiotiu et al., 2025). Key pathological features include goblet cell metaplasia, subepithelial fibrosis, angiogenesis, and ASM hypertrophy/hyperplasia, primarily orchestrated by cytokines such as IL-13, TGF-β, TSLP (Grainge and Park, 2018). TGF-β and IL-13 promote excessive extracellular matrix deposition and myofibroblast transformation (Camoretti-Mercado and Lockey, 2021; Grainge et al., 2014; Kraik et al., 2024; Mostaço-Guidolin et al., 2019; Willis et al., 2005). while epithelial-derived IL-25 upregulates VEGF/VEGFR to mediate angiogenesis (Corrigan et al., 2011). Halting this self-perpetuating structural transformation by targeting these molecular switches is paramount to improving long-term outcomes.

## 4 Methods

This study is presented as a structured narrative review due to substantial heterogeneity in study designs, interventions, dosing regimens, and outcome measures. A systematic literature search across the PubMed and Web of Science databases covering publications from January 2010 to June 2026. A combined search strategy of controlled vocabulary and free-text terms was adopted for retrieval. In PubMed, search fields included Title/Abstract and Medical Subject Headings (MeSH); in Web of Science, the “Topic” field (covering title, abstract, and keywords) was used. Search terms were categorized into three dimensions: (1) Disease dimension: “asthma” and related symptomatic terms; (2) Intervention dimension: “herbal medicine”, “herbal”, “medicinal plant”, etc.; (3) Component dimension: “phytochemical”, “plant extract”, etc. Relevant literature was identified through logical combinations of terms across these three dimensions.

**FIGURE 2:**
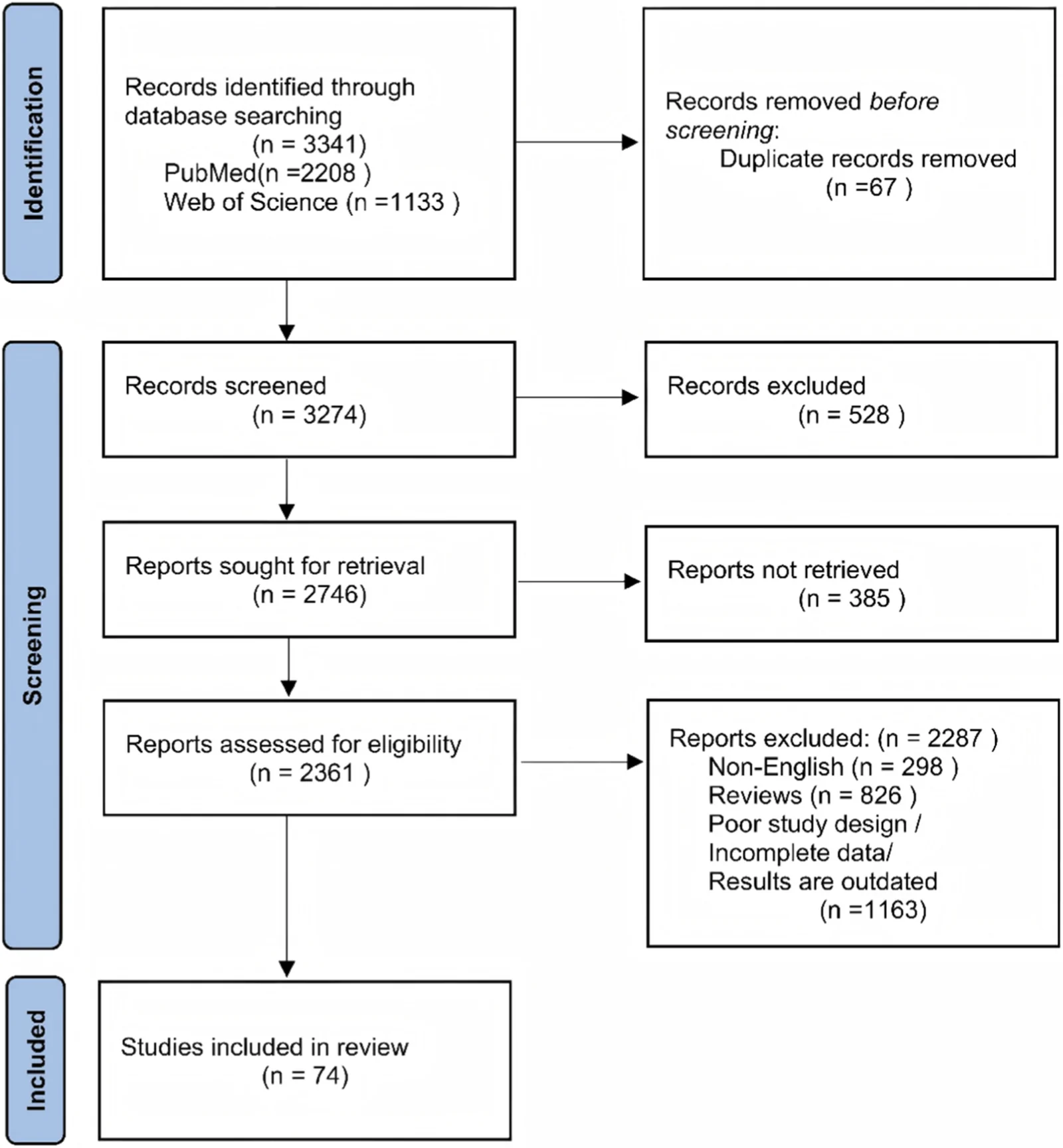
The PRISMA flowchart on the literature search procedure and selection of related studies.

Five independent researchers screened the retrieved records. Duplicate publications, non-English articles, and review papers were excluded. Full texts of the remaining articles were further evaluated, and studies irrelevant to the research objective, with incomplete methodological information, or lacking practical relevance in outcomes were removed. The literature selection process is illustrated in the PRISMA flow diagram Figure 2. Key findings of the included studies are summarized in Tables 1, 2, where upward (↑) and downward (↓) arrows denote the regulatory effects of phytochemicals on asthma-related parameters, respectively.

**TABLE 1:** Efficacy and mechanisms of botanical extracts in asthma models.

Extract types	Botanical	Model	*In vivo*/*in vitro*	Dosage/route of administration	Results	References
Perilla leaf extract	*Perilla frutescens* (L.) Britt.	*In vivo*: OVA *In vitro*: OVA Resistance DNP IgE + DNP-BSA	*In vivo*: Male BALB/c mice *In vitro*: PBMCs cells RBL-2H3 cells	*In vivo*:200 mg/kg oral gavage *In vitro*:50 μg/mL	↓EOS, ↓NEU ↓IL-6, ↓IL-4, ↓IL-4/IFN-γ, ↓TNF-α; ↓IL-8 ↓IgE, ↓IgG1, ↓IgG2a, ↓IgG2b ↓Syk, ↓p-Syk, ↓PKC, ↓p-PKC, ↓NF-κB p65 ↓p-NF-κB, ↓cPLA2, ↓p-cPLA2	Yang et al. (2020)
Saponin-enriched extract of Asparagus cochinchinensis	*Asparagus cochinchinensis* (Lour.) Merr	*In vivo*: OVA *In vitro*: LPS	*In vivo*: Female BALB/c mice *In vitro*: RAW264.7 cells	*In vivo*:250/500 mg/kg oral gavage *In vitro*:100/200 μg/mLl	↓EOS, ↓Mφ, ↓NO ↓iNOS mRNA, ↓COX-2 mRNA ↓IL-4mRNA, ↓IL-13mRNA ↓VEGF, ↓Collagen deposition	Sung et al. (2017)
Total flavonoids from Qu Zhi Qiao	*Citrus paradisi*Macfad. Cv. Changshanhuyou	*In vivo*: OVA	*In vivo*: Female/Male BALB/c mice	*In vivo*:25/50/100 mg/kg oral gavage	↓Raw, ↑Cdyn ↓WBC, ↓EOS, ↓NEU, ↓LYM, ↓MONO ↓α-SMA, ↓Cyclin D1 ↓TGF-β, ↓IFN-γ, ↓Eotaxin, ↓IL-4, ↓IL-5 ↓IL-13 ↓MUC5AC, ↓MUC5B; ↓MMP-2, ↓MMP-9 ↓p-Smad2, ↓p-Smad3, ↓p-p38, ↓p-Erk1/2	Wang J. et al. (2021)
Water extract and ethanol extract of Curcuma longa L.	*Curcuma longa* L.	*In vivo*: OVA	*In vivo*: Male Wistar rats	*In vivo*:0.75/1.5/3 mg/mL oral gavage (Add to drinking water)	↓WBC, ↓EOS, ↓NEU, ↓LYM ↓PLA2, ↓TP, ↓IgE ↓IL-4, ↓IFN-γ, ↓IFN-γ/IL-4 ↓MDA, ↓NO_2_, ↓NO_3_, ↑SOD, ↑CAT	Boskabady et al. (2021)
Ethanol extract of Curcuma longa L.	*Curcuma longa* L.	*In vivo*: OVA *In vitro*: TNF-α	*In vivo*: Male BALB/c mice *In vitro*: A549 cells	*In vivo*:100/400 mg/kg oral gavage *In vitro*:12.5/25/50 μM	↓Penh ↓IL-4, ↓IL-5, ↓IL-13, ↓IgE, ↓OVA IgE ↓p-ERK, ↓p-JNK, ↓p-p38 ↓ROS, ↓MDA, ↑SOD, ↑CAT, ↑GR, ↑GSH ↑IL-6, ↑TNF-α, ↑IL-1β mRNA ↓MMP-2, ↓MMP-9	Kim et al. (2024a)
Green Tea Extract	*Camellia sinensis* (L.) Kuntze	*In vivo*: OVA	*In vivo*: Male BALB/c mice *In vitro*: NCI-H292 cells	*In vivo*:100/400 mg/kg/day oral gavage *In vitro*: GTE 5–160 μg/mL	↓Penh, ↓ERK/JNK/p38 ↓MMP-9, ↓MDA, ↑GSH, ↑CAT, ↑SOD ↓TNF-α ↓IL-1β, ↓IL-6 mRNA	Kim et al. (2024b)
Water extract of Zingiber officinalisroscoe L.	*Zingiber officinalis*Roscoe	*In vivo*: OVA	*In vivo*: Male Wistar rats	*In vivo*:207/414 mg/kg oral gavage	↓WBC, ↑RBC ↓ASAT, ↓CRP ↓MDA, ↓AOPP, ↓GPx, ↓GSH	Jedli et al. (2022)
Cimicifugae Rhizoma Extract	*Cimicifuga racemosa* (L.) Nutt.	*In vivo*: OVA	*In vivo*: Female BALB/c mice	*In vivo*:30/100 mg/kg oral gavage	↓Penh ↓IL-4, ↓IL-5, ↓IL-13, ↓OVA IgE ↑HO-1, ↑Nrf2, ↑NQO1 ↓MMP-9, ↓NF-κB, ↓p-NF-κB	Lim et al. (2021)
Undaria pinnatifida extract	*Undaria pinnatifida* (Harv.) Suringar	*In vivo*: OVA	*In vivo*: Male BALB/c mice	*In vivo*:50/100/200 mg/kg/day oral gavage	↓EOS, ↓NEU, ↓LYM, ↓Mφ ↓OVA IgE, ↓IgG1, ↑IgG2a ↓IL-4, ↓IL-5, ↓IL-13, ↑IFN-γ; ↓IL-33, ↓ST2 ↓MDA, ↑SOD, ↑HO-1, ↑Nrf2 ↑E-cadherin, ↑ZO-1, ↑Occludin ↓NF-κB, ↓p-NF-κB, ↓p38, ↓Erk, ↓JNK	Yu Z. N. et al. (2024)
Cade oil	*Juniperus oxycedrus* L.	*In vivo*: OVA	*In vivo*: Male Wistar rats	*In vivo*:1 mL/kg oral gavage	↓LYM, ↓MONO ↓MDA, ↑GSH, ↑GPx, ↑CAT	Ahmida et al. (2024)
Leonurus sibiricus	*Leonurus sibiricus* L.	*In vitro*: HRV16	*In vitro*: WI-38 cells HFL1 cells	*In vitro*:0.156/0.312/0.625 mg/mL	↓MMP-9`↓Arginase I,↓TGF-β	Wieczfinska et al. (2020)
Water extract and ethanol extract of Salvia miltiorrhizaBunge	*Salvia miltiorrhiza* Bunge	*In vivo*: OVA *In vitro*: TGF-β1	*In vivo*: Female BALB/c mice *In vitro*: BEAS-2B cells MRC-5 cells	*In vivo* WE:31.2/156 mg/kg EE:49.2/246 mg/kg oral gavage *In vitro*: (Key Ingredients) Salvianic acid A:10/100 μM Caffeic acid:10/100 μM Rosmarinic acid: 10/100 μM Salvianolic acid B: 10/100 μM Tanshinone IIA:10/100 μM	↓OVA IgE ↓ EOS, ↓NEU ↓IL-4, ↓IL-5, ↓IL-13, ↑IFN-γ Caffeic acid/Rosmarinic acid ↓E-cadherin, ↓vimentin mRNA ↓α-SMA, ↓COL1A1 mRNA	Luo et al. (2019)
Agarwood Nanoemulsion	*Aquilaria sinensis* (Lour.) Spreng.	*In vitro*: TGF-β1	*In vitro*: BEAS-2B cells	*In vitro*:25 μg/mL	↑NO, ↓MMP-9, ↓Angiogenin, ↓PTX3	Malik et al. (2026)
M. longifoliasubsp. Schimperi	*Mentha longifolia* (L.)	*In vivo*: OVA	*In vivo*: Male Swiss albino mice	*In vivo*:25 mg/kg oral gavage	↓EOS, ↓NEU, ↓LYM, ↓MONO, ↓BAS ↑GSH, ↓Nox; ↓LDH, ↓TPC	Haikal et al. (2025)
Water extract of Sophora flavescens Aiton	*Sophora flavescens* Aiton	*In vivo* OVA + PM *In vitro* TNF-α/IL-4	*In vivo*: BALB/c mice *In vitro*: BEAS-2B cells	*In vivo*:22/265 mg/kg oral gavage *In vitro*:100 μg/mL	↓GM-CSF, ↓IL-6, ↓IL-8, ↓MCP-1 ↓ROS, ↓γ-H2AX ↓ERK, ↓JNK	Park et al. (2026)
Agarwood oil	*Aquilaria sinensis* (Lour.) Spreng.	*In vitro*: LPS	*In vitro*: RAW264.7 cells	*In vitro*:25/50 μg/mL	↓ROS, ↓NO, ↓iNOS mRNA, ↓HO-1 mRNA ↓TNF-α, ↓IL-6, ↓IL-1β mRNA	Malik et al. (2023)

**TABLE 2:** Efficacy and mechanisms of isolated phytochemicals in asthma models.

Chemical composition	Model	*In vivo*/*in vitro*	Dosage/Route of administration	Results	References
Linarin	*In vivo*: HDM *In vitro*: HDM	*In vivo*: Female BALB/c mice *In vitro*: BEAS-2B cells	*In vivo*:30/60 mg/kg Intraperitoneal injection *In vitro*:50 μM	↓EOS, ↓IL-4, ↓IL-5,↓IL-13, ↓IL-4, ↑IFN-γ, ↓IgE ↓mtROS, ↓Ser616, ↑MFN2 ↓cGAS-STING ↑GPX4, ↑xCT, ↑FHC, ↓PTGS2, ↓FACL4	Ding et al. (2025a)
Linarin	*In vivo*:OVA	*In vivo*: Male Wistar rats	*In vivo*:50 mg/kg oral gavage	↓WBC, ↓EOS, ↓NEU ↓histamine, ↓MDA, ↑SOD, ↑CAT; ↑GSH ↓IL-4, ↓IL-13.↓IgE, ↑IFN-γ, ↓TNF-α	Shen et al. (2025)
Quercetin	*In vivo*: OVA	*In vivo*: Male Wistar rats	*In vivo*:50 mg/kg	↑TAC↑SOD, ↑GPX, ↑CAT, ↓MDA ↓IL-6, ↓TNF-α, ↑IL-10 ↓GATA-3, ↓α-SMA, ↓IL-1β, ↓TGF-β, ↑T-bet	Rajizadeh et al. (2023)
Quercetin	*In vivo*: OVA	*In vivo*: Female BALB/c mice	*In vivo*:40/80 mg/kg Intraperitoneal injection	↓IL-4, ↓IL-5, ↓IL-13 ↓ROS, ↓MDA, ↑SOD ↑Nrf2, ↑SIRT1, ↑NQO1	Sun et al. (2024)
Quercetin	*In vitro*:IL-13	*In vitro*: HBECs	*In vitro*:25 μM	↑Nrf2; ↓TNF-α ↑GCLC, ↑NQO1	Winnica et al. (2025)
Artemetin	*In vivo*: HDM *In vitro*: HDM	*In vivo*: Female BALB/c mice *In vitro*: BEAS-2B cells	*In vivo*:50/100 mg/kg Intraperitoneal injection *In vitro*:50 μM	↓EOS, ↓IL-4, ↓IL-5, ↓IL-13.↓IgE ↑CD4^+^IFN-γ^+^, ↓CD4^+^IL-4^+^ ↓MDA, ↑SOD, ↑CAT; ↑Nrf2/HO-1 ↓mtROS, ↑MMP; ↓p-DRP1, ↑MFN2 ↓GSDMD, ↓Caspase-8,↓BAX, ↓RIPK1, ↓FADD, ↑Bcl-2	Ding et al. (2025b)
Xanthohumol	*In vivo*: OVA	*In vivo*: Female BALB/c mice	*In vivo*:15/30/60 mg/kg oral gavage	↓IL-4, ↓IL-5, ↓IL-13, ↓IgE, ↑Treg ↓ROS, ↑SOD, ↑GSH ↓AIM2, ↓ASC, ↓caspase-1, ↓IL-1β,↓IL-18	Chen et al. (2026)
Tectorigenin	*In vivo*: OVA	*In vivo*: Male BALB/c mice	*In vivo*:10/25 mg/kg	↑IL-12, ↑T-bet, ↓IL-4, ↓IL-5, ↓IL-13, ↓GATA3 ↓TNF-α, ↓IL-6, ↓TGF-β ↑Nrf2, ↑HO-1↓, Keap1, ↓ROS, ↓MDA ↓α-SMA.↑E-cadherin	Jiang et al. (2024)
Baicalin	*In vivo*: OVA *In vitro*: PDGF-BB	*In vivo*: Female C57BL/6 mice *In vitro*: SMC cells	*In vivo*:50 μg/g oral gavage *In vitro*:0.1 mg/mL	↓MDA, ↓NO, ↑SOD, ↓IL-6, ↓TNF-α ↑miR-103, ↓p-NF-κB,↓p65	Zhai and Wang (2022)
Wogonoside	*In vivo*: OVA *In vitro*:IL-13	*In vivo*: Female BALB/c mice *In vitro*:16HBE cells	*In vivo*:10/20 mg/kg oral gavage *In vitro*:25/50/100 μM	↓sRaw, ↓EOS, ↓LYM ↓IL-4, ↓IL-5, ↓IL-13, ↓IgE, ↓TNF-α, ↓IL-6 ↓TGF-β ↓MUC5AC, ↓MUC5B, ↓GOB5 ↓p-p65, ↓p-STAT6	Yu X. et al. (2024)
Formononetin	*In vivo*: OVA	*In vivo*: Female BALB/c mice	*In vivo*:10/20/40 mg/kg	↓RL, ↓NEU, ↓LYM ↓IL-4, ↓IL-5, ↓IL-13, IL-17A, ↓IgE, ↓CCL5 ↓CCL11 ↓ROS, ↑SOD ↓NF-κB, ↓JNK, ↑SOD1, ↑HO-1	Yi et al. (2020)
Breviscapine	*In vitro*: LPS	*In vitro*: NHBE cells	*In vitro*:10/20/40 μg/mL	↓IL-1β, ↓IL-6, ↓MCP-1 ↓MDA, ↑SOD, ↑GSH-Px ↓MUC5AC mRNA; ↓Col-I`FN mRNA ↓TLR4, ↓MyD88, ↓TRAF6	Zhao et al. (2025)
Neohesperidin	*In vivo*: OVA	*In vivo*: Female BALB/c mice *In vitro*: BEAS-2B cells	*In vivo*:20 mg/kg Intraperitoneal injection *In vitro*:20 μM	↓Penh, ↑IFN-γ`↑IL-12, ↓IgE,↓IgG1, ↑IgG2a ↓TNF-α ↓IL-4, ↓IL-5, ↓IL-13 ↓MDA, ↑GSH, ↑CAT, ↑SOD, ↓COX-2, ↑HO-1 ↓IL-6, ↓IL-8`↓CCL5, ↓MCP-1`↓CCL11 ↓CCL24, ↓ROS	Cheng et al. (2025)
Naringenin	*In vivo*: OVA	*In vivo*: Male Wistar rats	*In vivo*:20/40 mg/kg oral gavage	↓IL-4, ↓IL-13; ↓EOS ↓MDA, ↑GSH ↓UCN, ↓SP-D	Jasemi et al. (2022)
Icariin	*In vivo*: OVA *In vitro*: TNF-α	*In vivo*: Female BALB/c mice *In vitro*: HBE135-E6E7 cells	*In vivo*:25/50/100 mg/kg oral gavage *In vitro*:25/50/100 μM	↓RL, ↑Cdyn ↓ROS, ↓MDA, ↑SOD, ↑CAT ↑Nrf2, ↓Keap-1 ↓p-p65, ↓p-IκBα, ↓p-Iκα/β; ↓iNOS ↓IL-4, ↓IL-5, ↓IL-13, ↓TNF-α, ↓IgE;, ↓IL-17A	Wang et al. (2026)
Icariin	*In vivo*: OVA + RSV	*In vivo*: Female BALB/c mice	*In vitro*:10/20/40 μg/mL	↓EOS, ↓NEU, ↓Mφ ↓IL-4, ↓IL-5, ↓IL-13 ↓MDA, ↑SOD, ↑CAT, ↑GSH, ↑Nrf2/HO-1 ↓Bax, ↓Cleaved caspase-3, ↑Bcl-2	Fu and Wang (2025)
Epimedin C	*In vivo*: OVA	*In vivo*: Female BALB/c mice	*In vivo*:20/40/80 mg/kg	↓RL, ↑Cdyn ↓WBC, ↓EOS, ↓NEU, ↓LYM, ↓MONO ↓IL-4, ↑IL-10 ↓p52, ↓RelB, ↓p-ERK1/2, ↓p-p38 MAPK	Huang et al. (2020)
Luteolin	*In vivo*: OVA	*In vivo*: Female BALB/c mice	*In vivo*:10/20 mg/kg	↑Cdyn, ↓EOS, ↓NEU, ↓LYM ↓IL-4, ↓IL-5, ↓IL-13, ↓IgE ↓LC3B, ↑p62; ↓Beclin-1, ↓PI3KC3 ↑PI3K p85, ↑p-Akt, ↑p-mTOR	Wang S. et al. (2021)
Tectorigenin	*In vivo*: OVA	*In vivo*: Male Guinea pigs	*In vivo*:10/25 mg/kg oral gavage	↓Penh, ↓EOS, ↓NEU, ↓Mφ↓, LYM ↓IL-1β, ↓IL-4, ↓IL-6, ↓IL-8, ↓IL-13 ↓TGF-β1, ↓p-Smad2/3, ↓Smad4, ↑Smad7 ↓TLR4, ↓MyD88, ↓p-IKKβ, ↓NF-κB ↓VEGFA, ↓TNF-α, ↑IFN-γ	Wang et al. (2020)
Myricetin	*In vivo*: OVA *In vitro*: LPS	*In vivo*: Mice (Not specified) *In vitro*:3T6 cells RAW264.7 cells Joint cultivation	*In vivo*:100/200 mg/kg Intraperitoneal injection *In vitro*:20/40/60 μM	↓IL-5, ↓IL-6, ↓IL-1β ↑Sirt1, ↓α-SMA ↓p-JNK, ↓p-Smad3, ↓ace-Smad3	Huang et al. (2024b)
Apigenin	*In vivo*: HFD + OVA *In vitro*: HDM	*In vivo*: Male C57BL/6 mice *In vitro*:16HBE cells	*In vivo*:10/20 mg/kg oral gavage *In vitro*:20 μM	↓WBC, ↓EOS, ↓NEU, ↓LYM ↓MPO,↓IL-4,↓IL-5, ↓IL-13.↓IgE, ↓IFN-γ ↓IL-17 ↓T-bet, ↓Gata-3, ↓RORyt, ↑Foxp3 ↓ROS, ↓p-ASK1, ↓p-JNK, ↓p-p38 ↓Cytochrome c, ↓Bax, ↓cleaved-caspase-3 ↑Bcl-2	Yu et al. (2023)
Sophoraflavanone G	*In vivo*: OVA	*In vivo*: Female BALB/c mice *In vitro*: BEAS-2B cells	*In vivo*:5/10 mg/kg Intraperitoneal injection *In vitro*:IL-4/TNF-α(10 ng/mL)	↓Penh,↓EOS ↓MDA, ↓NO, ↑GSH, ↑CAT ↓IL-4, ↓IL-5, ↓IL-13, ↓TNF-α, ↓IL-6, ↓CCL24. ↑IFN-γ ↓IgE,↓IgG1,↑IgG2a	Wang et al. (2022)
Calcaratarin D	*In vivo*: HDM	*In vivo*: BALB/c mice	*In vivo*:12.5/25/50 mg/kg oral gavage	↓EOS, ↓Mφ, ↓NEU ↓RI, ↑Cdyn; ↓IL-4, ↓IL-5, ↓TNF-α, ↓IL-6 ↓4-HNE, ↓8-OHdG; ↑SOD, ↑CAT, ↓Arg1 ↓JAK1/STAT6, ↓FoxO1	Liao et al. (2023)
Involucrasin B	*In vivo*: HFD + HDM *In vitro*: LPS/PA	*In vivo*: Male C57BL/6 mice *In vitro*: THP-1 cells	*In vivo*:50/100 mg/kg oral gavage *In vitro*:15/30/60 μM	↓LDL-C, ↓T-CHO, ↓TG, ↑HDL-C; ↓Penh ↓IL-1β, ↓IL-6, ↓IL-8, ↓IL-17A, ↓IL-18 ↓IL-22, ↓TNF-α ↓GM-CSF; ↓Gr-1, ↓NETs; ↓ILC3; ↓Th17 ↓NLRP3, ↓ASC, ↓Caspase-1, ↓ iNOS ↓p-p65,↓p65	Yang et al. (2024)
Cycloastragenol	*In vivo*: OVA	*In vivo*: Female BALB/c mice	*In vivo*:31.25/62.5/125 mg/kg	↑Cydn, ↓EOS ↓IL-5, ↓IL-13.↓IgE ↓LC3B-II/LC3B-I, ↑p62, ↓Beclin-1	Zhu et al. (2021)
Astragaloside IV	*In vitro*: TNF-α	*In vitro*: BEAS-2B cells	*In vitro*:0–100 μM	↓IL-6, ↓IL-8, ↓MCP-1, ↓CCL5 ↓ICAM-1; ↓ROS ↓IκB-α, ↓p65, ↓ERK1/2, ↓JNK, ↓p38	Hsieh et al. (2022)
Ginsenoside Rg3	*In vivo*: OVA *In vitro*: TNF-α/IL-4	*In vivo*: Female BALB/c mice *In vitro*: BEAS-2B cells	*In vivo*:5/10 mg/kg Intraperitoneal injection *In vitro*:0–30 μM	↓Penh, ↓Airway resistance; ↓EOS ↓IL-4, ↓IL-5, ↓IL-13, ↓CCL24, ↓CCL11, ↑IFN-γ, ↓IgE, ↑IgG1, ↑IgG2a, ↓IL-8, ↓MCP-1, ↓CCL5 ↓ICAM-1 ↓MDA, ↑SOD, ↑GSH; ↑Nrf2/HO-1; ↓NF-κB	Huang et al. (2021)
Betulin	*In vivo*: OVA	*In vivo*: Female BALB/c mice	*In vivo*:10 mg/kg Intraperitoneal injection	↓ROS, ↑SOD, ↑CAT, ↑GSH, ↓NO_2_^−^, ↓NO_3_^−^ ↓MDA ↓IL-4, ↓IL-5, ↓IL-13, ↓TNF-α, ↓IgE;, ↑IFN-γ ↓tTG, ↓TGF-β1, ↓MMP-9 ↓TREM-1, ↓p-IκB-α↓NF-κBp65	Kamaraj et al. (2021)
Platycodin D	*In vivo*: HFD + OVA *In vitro*:IL-13	*In vivo*: Female C57BL/6 mice *In vitro*: BEAS-2B cells	*In vivo*:20 mg/kg Intraperitoneal injection *In vitro*:0–10 μM	↓IL-6, ↓TGF-β, ↓TNF-α ↓ROS, ↑SOD, ↓MDA; ↓RI ↓IL-4, ↓IL-5, ↓IL-13, ↓IL-17, ↓CCL11	Xu et al. (2023)
Curcumin	*In vivo*: OVA	*In vivo*: BALB/c mice (Gender not specified)	*In vivo*:10 mg/kg Intraperitoneal injection	↓EOS, ↓NEU; ↓ROS, ↓NO ↓IL-5, ↓IgE ↓MMP-9, ↓α-SMA ↓HDAC8, ↓NF-κB, ↓p-p38 MAPK, ↓p-ERK ↓p-JNK	Islam and Singh (2023)
Curcumin	*In vivo*: OVA *In vitro*: DBP	*In vivo*: BALB/c mice (Gender not specified) *In vitro*: A549 cells	*In vivo*:5 mg/kg/Intranasal medication *in vitro*:25 μM	↓ROS, ↓NO, ↓MDA; ↑GSH.↑SOD.↑GPx ↓MMP-9 ↑Nrf2, ↑GPx4, ↑SLC7A11, ↓SLC40A1 ↓NCOA4, ↓FTH-1 ↓ACSL4, ↓FTH-1, ↑GPx-4 ↓8-oxodG, ↓PARP-1	Singh et al. (2026)
Rosmarinic Acid	*In vivo*: OVA	*In vivo*: Female BALB/c mice	*In vivo*:2.5/5/10 mg/kg	↓WBC, ↓EOS, ↓LYM, ↓BAS ↓IL-4, ↓IL-5, ↓IL-13 ↑Short-chain fatty acid-producing bacteria ↓Lipopolysaccharide-producing bacteria ↑ZO-1, ↑OCLN; ↓MCP-1, ↓TNF-α, ↓IL-1β	Guo et al. (2024)
Rosmarinic Acid	*In vivo*: OVA	*In vivo*: Male Wistar rats	*In vivo*:0.5/1/2 mg/kg oral gavage	↓IL-4, ↓IL-17A, ↑IFN-γ ↓MDA, ↓NO, ↑CAT, ↑SOD, ↑SH	Abbasnia et al. (2026)
Rosmarinic Acid	*In vivo*: OVA	*In vivo*: Male Wistar rats	*In vivo*:0.5/1/2 mg/kg oral gavage	↓WBC, ↓EOS, ↓NEU, ↓MONO ↓MDA, ↓NO_2_, ↑SOD, ↑CAT	Abbasnia et al. (2024)
Resveratrol	*In vivo*: OVA *In vitro*: LPS	*In vivo*: Female BALB/c mice *In vitro*: MLE-15 cells	*In vivo*:100 mg/kg oral gavage *In vitro*:50 μM	↑sRaw, ↓dT, ↑PEF, ↓Ti/Te ↑occludin, ↑claudin, ↑ZO-1, ↓MUC5ac ↓CCL-2, ↓IL-13, ↓IL-1β, ↓IL-4, ↓IL-5, ↓IFN-γ ↓TNF-α, ↑IL-10	Alharris et al. (2022)
Oleuropein, OLP	*In vitro*:IL-17	*In vitro*: A549 cells	*In vitro*:10–100 ng/mL	↓ROS, ↓JC-1; ↓γH2AX	Montalbano et al. (2024)
Myrtenol	*In vivo*: OVA	*In vivo*: Male Wistar rats	*In vivo*:8 mg/kg Nebulization	↓TNF-α; ↓IL-6,↓Th17; ↑IL-10 Hippocampus↓MDA, ↑SOD, ↑GPx	Bejeshk et al. (2023)
Myrtenol	*In vivo*: OVA	*In vivo*: Male Wistar rats	*In vivo*:50 mg/kg Intraperitoneal injection	↓MDA, ↑SOD ↓TNF-α, ↓IL-1β, ↑IFN-γ, ↑IL-10	Bejeshk et al. (2019)
Paeoniflorin	*In vivo*: OVA	*In vivo*: Female C57BL/6 mice	*In vivo*:10/25/50 mg/kg Nebulization	↓IL-5, ↓IL-9, ↓IL-25, ↓IL-33, ↓IgE ↓Th2, ↓Th17; ↑Th1,↑Treg ↓ROS, ↑MMP ↓Beclin-1, ↓p62, ↓Bax, ↓cleaved caspase-3 ↑Bcl-2	Han et al. (2022)
Artesunate	*In vivo*: HDM	*In vivo*: Female BALB/c mice	*In vivo*:30 mg/kg Intraperitoneal injection	↓FIZZ1, ↓p-p38 MAPK; ↓Airway resistance ↓IL5RA, ↓CCR3, ↓IL13RA2, ↓TNFRSF8 mRNA	Zhang M. et al. (2023)
Sinomenine	*In vivo*: HFD + OVA *In vitro*: LPS	*In vivo*: Female BALB/c mice *In vitro*:16HBE cells	*In vivo*:35/75 mg/kg oral gavage *In vitro*:200 μg/mL	↓Vimentin, ↓MMP7, ↓MMP9 ↓IgE, ↓IL-4; ↓TGF-β1, ↓Smad3	He et al. (2021)
Falcarindiol	*In vivo*: OVA *In vitro*:IL-13	*In vivo*: Male BALB/c mice *In vitro*: BEAS-2B cells	*In vivo*:100/200 mg/kg oral gavage *In vitro*:5/10/20 μM	↓WBC, ↓EOS ↓IL-4, ↓IL-5, ↓IL-13, ↓IgE ↓MDA, ↑SOD, ↑CAT; ↑GSH-Px ↑Nrf2, ↑HO-1, ↑NQO1	Jiang et al. (2026)
Gentisic acid	*In vivo*: OVA	*In vivo*: Female BALB/c mice	*In vivo*:50/100 mg/kg oral gavage	↓WBC, ↓EOS, ↓NEU,↓Mφ ↓IgE; ↓MDA, ↑GSH, ↑CAT, ↑GPx ↑Nrf2/HO-1, ↓NF-κB	Abdelmawgood et al. (2025)
Mulberroside A	*In vivo*: OVA/OVA + LPS *In vitro*: TNF-α/IL- 4+TNF-α	*In vivo*: Female BALB/c mice *In vitro*: BEAS-2B cells THP-1 cells	*In vivo*:10/20 mg/kg 20 mg/kg Intraperitoneal injection *In vitro*:5/10/20/30 μM	↓Penh,↓Airway resistance; ↓EOS, ↓NEU ↓IL-4, ↓IL-5, ↓IL-13, ↓, TNF-α′IgE; ↑IFN-γ ↑IL-12 ↓IL-6, ↓IL-8, ↓CCL5, ↓MCP-1, ↓CCL11, ↓CCL24 ↓ICAM-1 ↓ROS, ↓MDA, ↑SOD, ↑CAT, ↑GSH; ↑Nrf2/HO-1 ↓OVA-IgE, ↓IgG1, ↑IgG2a, ↓Ly6G	Liou et al. (2025)
Stigmasterol	*In vivo*: OVA *In vitro*:IL-13	*In vivo*: Male C57BL/6 mice *In vitro*: BEAS-2B cells	*In vivo*:100 mg/kg Intraperitoneal injection *In vitro*:10/20 μg/mL	↓Airway resistance,↓EOS ↓IL-4, ↓IL-5, ↓IFN-γ ↓MDA, ↑SOD, ↑CAT; ↑NK1R	Zhang J. et al. (2023)
Auraptene	*In vitro*: LTA	*In vitro*: RAW264.7 cells	*In vitro*:5/10 μM	↓NO, ↓iNOS ↓IL-1β, ↓TNF-α, ↓COX-2 ↓ERK1/2, ↓JNK1/2, ↓IκBα, ↓p65	Hsia et al. (2021)
Arctiin	*In vivo*: OVA	*In vivo*: Female C57BL/6 mice	*In vivo*:10 mg/kg Intraperitoneal injection	↓NEU, ↓EOS, ↓LYM ↑IFN-γ, ↓IL-4, ↓IL-5 ↑PEF.↑Cdyn,↓RAW; ↑SOD; ↑GSH ↓p-p38/p38, ↓p-NF-κB p65	Yuan and Sun (2024)
Schisandrin A	*In vivo*: HFD + OVA *In vitro*: HDM/LPS	*In vivo*: Male BALB/c mice *In vitro*:16HBE cells/RAW264.7 cells	*In vivo*:40 mg/kg Intraperitoneal injection *In vitro*:1/5/10 μM	↓EOS, ↓NEU, ↓LYM, ↑Mφ ↓α-SMA ↓iNOS, ↓COX-2, ↓NF-κB p65	Qiu et al. (2023)
Cinnamaldehyde	*In vivo*: OVA	*In vivo*: Female BALB/c mice	*In vivo*:20/40 mg/kg	↓WBC, ↓EOS, ↓LYM, ↓MONO ↓IgE, ↓IL-13; ↓NF-κB p65 ↓MDA, ↓NO, ↑GSH, ↑CAT	Nour et al. (2026)
Isorhapontigenin	*In vivo*: OVA *In vitro*: TNF-α/IL-4	*In vivo*: Female BALB/c mice *In vitro*:BEAS-2B cells	*In vivo*:30 mg/kg oral gavage *In vitro*:0–50 μM	↓RI, ↑Cdyn,↓Penh ↓IL-4, ↓IL-5, ↓IL-13, ↓TNF-α, ↓IL-6, ↓TGF-β ↑IFN-γ, ↑IL-12; ↑SOD, ↑CAT, ↑GSH,↑HO-1 ↓MDA ↓COX-2, ↓IgE, ↓IgG1, ↑IgG2a	Huang et al. (2026)
Plumbagin	*In vivo*: OVA + HFD	*In vivo*: Male C57BL/6 mice	*In vivo*:1 mg/kg Intraperitoneal injection	↓EOS ↓TNF-α, ↓IL-4, ↓IL-5, ↓eotaxin ↓ROS, ↑SOD; ↑p-AMPK	Zhang L. et al. (2023)
Salidroside	*In vivo*: OVA	*In vivo*/Male BALB/c mice	*In vivo*:48 mg/kg/day oral gavage	↓IL-4, ↓IL-5, ↓IL-13, ↑IFN-γ, ↑IL-10, ↑SOD ↑GSH-Px, ↓MDA, ↓α-SMA`↓MMP2/9`↓TGF-β_1_	Wang et al. (2023)
Cordycepin	*In vivo*: OVA	*In vivo*: Female Wistar rats	*In vivo*:50 mg/kg	↓EOS,↓NEU ↓IL-5,↓IL-13,↓IgE,↓TNF-α ↓TGF-β_1_,↑A2AAR mRNA,↓p38MAPK	Fei et al. (2017)
Cordyceps militaris polysaccharide	*In vivo*: OVA	*In vivo*: Male BALB/c mice	*In vivo*:50/100/200 mg/kg oral gavage	↓IgE, ↑IFN-γ, ↓TNF-α, ↓IL-4, ↓IL-5, ↓IL-6 ↓IL-13 ↑Nrf2`↑HO-1`↑SOD1/2 mRNA ↓NF-κB`↓IκBα`↓IKKα/β mRNA ↓P-P65	Song et al. (2023)
Ephedrae Botanical druga polysaccharides	*In vivo*: OVA	*In vivo*: Male SD rats	*In vivo*:137.71/275.42 mg/kg oral gavage	↓TGF-β_1_, ↓IL-6, ↑IL-10 ↓IgE, ↓TNF-α, ↓IL-4, ↑IFN-γ ↓IL-4mRNA, ↓TGF-β_1_mRNA, ↓Il-6mRNA ↓TNF-α mRNA, ↓IL-13mRNA; ↑IFN-γ mRNA` ↑IL-10mRNA ↓ROS, ↓MDA, ↑T-SOD, ↑GSH-Px ↓Mφ, ↓NEU; ↑CD4^+^CD8a^+^, ↑NK; ↑DCs ↑Treg, ↓Th17, ↓IL-17A	Zhang et al. (2022)

## 5 Results

A total of 3,341 records were identified through database searching. Following deduplication, 67 studies were removed. During the title and abstract screening phase, 528 studies were excluded, and an additional 385 studies were excluded due to unavailable full texts, leaving 2,361 articles for full-text assessment. During the full-text screening phase, 298 non-English publications were excluded, 826 reviews were removed, and 1,163 studies were discarded due to issues regarding scientific rigor of experimental design, data integrity, or timeliness of reported outcomes. Ultimately, 74 articles were included in this review.

## 6 Botanical extracts

Botanical extracts are key raw materials for drug development, obtained through physical or chemical methods from whole plants or specific parts to target active ingredients without destroying their original chemical structure. Compared to single compounds, whole botanical extracts usually contain multiple synergistic active ingredients, demonstrating multi-target, multi-pathway pharmacological advantages in asthma treatment.

Various botanical extracts exert effects by inhibiting key inflammatory pathways and regulating Th1/Th2 balance. *Perilla frutescens(L.)* Britt [Lamiaceae; Perillae folium] leaf extract contains roseoside (RosS), vicenin-2 (Vic-2), and rosmarinic acid (RosA), which synergistically target Syk kinase, inhibiting downstream PKC/NF-κB/cPLA_2_ pathways to alleviate airway inflammation (Yang et al., 2020). Saponin-enriched extract of *Asparagus cochinchinensis(Lour.)* Merr [Asparagaceae; Asparagi cochinchinensis radix] (SEAC) exerts efficacy by downregulating COX-2, iNOS, and Th2 cytokines (IL-4, IL-13), and reducing IgE and VEGF generation (Sung et al., 2017). Total flavonoids from *Citrus paradisi* Macfad. Cv. Changshanhuyou [Rutaceae; Citri grandis exocarpium] (TFCH) dose-dependently inhibit MAPKs and Smad2/3 pathways, effectively reducing inflammatory infiltration, lowering Th2 factor levels, and inhibiting collagen deposition, with high-dose TFCH (100 mg/kg) tends to approximate the improvement magnitude achieved by dexamethasone (DEX) across most indicators, while still showing divergence in metrics such as p-Erk1/2 (Wang et al., 2021a). *Curcuma longa*L. [Zingiberaceae; Curcumae longae rhizoma] extract (CLE) not only inhibits Th2 cytokines and serum IgE but also elevates the IFN-γ/IL-4 ratio, showing potential in correcting Th1/Th2 imbalance (Kim et al., 2024a; Boskabady et al., 2021).

Targeting oxidative stress mechanisms, *Camellia sinensis* (L.) Kuntze [Theaceae; Camelliae sinensis folium] (green tea) extract inhibits the MAPKs/MMP-9 pathway, alleviating OVA-induced allergic asthma symptoms (Kim et al., 2024b). *Zingiber officinale* Roscoe [Zingiberaceae; Zingiberis rhizoma] water extract reduces MDA and AOPP levels, increases GPx and GSH activities, and inhibits the STAT6/TNF-α pathway, reducing inflammatory damage, oxidative stress, and blood biochemical disturbances (Jedli et al., 2022). *Cimicifuga racemosa* (L.) Nutt. [Ranunculaceae; Cimicifugae rhizoma] ethanol extract exerts antioxidant effects by activating the Nrf2/HO-1/NQO1 pathway while inhibiting the NF-κB/MMP-9 pathway to reduce inflammation, possibly related to its phenolic acid content (Niu et al., 2019; Lim et al., 2021). *Undaria pinnatifida* (Harv.) Suringar [Phaeophyceae; Undariae pinnatifidae thallus] extract (UPE) elevates SOD, Nrf2, and HO-1 activities, reduces MDA levels, upregulates ZO-1, Occludin, and E-cadherin expression, and inhibits NF-κB/MAPK signaling pathway activation and the IL-33/ST2 axis, thereby exerting anti-inflammatory, antioxidant, and airway barrier repair functions (Yu et al., 2024b). Cade oil (*Juniperus oxycedrusL.* [Cupressaceae; Juniperi oxycedri lignum]), rich in polyphenols and flavonoids, significantly improves asthma symptoms by scavenging ROS, inhibiting inflammatory cell recruitment, and enhancing endogenous antioxidant enzyme activities (Ahmida et al., 2024).

In reversing airway remodeling, *Leonurus sibiricus*L [Lamiaceae; Leonuri herba] root extract slows the progression of subepithelial fibrosis by inhibiting key factors like arginase I, MMP-9, and TGF-β (Wieczfinska et al., 2020). *Salvia miltiorrhiza*Bunge [Lamiaceae; Salviae miltiorrhizae radix et rhizoma] water extract, rich in caffeic acid and rosmarinic acid, performs excellently in inhibiting the TGF-β_1_ pathway, showing superior effects to ethanol extract in improving airway reactivity and reversing remodeling (Luo et al., 2019). Agarwood (*Aquilaria sinensis* (Lour.) Spreng. [Thymelaeaceae; Aquilariae lignum]) nanoemulsion (AW-NE) shows potential to delay airway remodeling by restoring NO production, inhibiting excessive epithelial migration, and downregulating remodeling-related proteins like MMP-9 and angiogenin, though its efficacy awaits further validation *in vivo* (Malik et al., 2026).

*Mentha longifolia* (L.) Hudson subsp. *schimperi* (Briq.) Greuter & Burdet [Lamiaceae; Menthae herba] whole extract and its flavonoids regulate GSH and NO_x_ levels, significantly reducing oxidative stress and inflammatory cell infiltration *in in vivo* models (Haikal et al., 2025). Particulate matter (PM) induces ROS bursts, activates MAPK signaling, disrupts autophagic flux, leading to airway inflammation and cell damage. The traditional botanical drug *Sophora flavescens* Aiton [Fabaceae; Sophorae flavescentis radix] alleviates PM-exacerbated asthma inflammation by scavenging PM-induced ROS bursts, downregulating JNK phosphorylation, and partially restoring autophagic balance (Park et al., 2026). Additionally, agarwood (*Aquilaria sinensis* (Lour.) Spreng. [Thymelaeaceae; Aquilariae lignum]) oil nano-lipid emulsion (DE'RAQSIN, a prepared formulation of agarwood oil) blocks the TLR4/NF-κB pathway, reduces ROS/NO production and pro-inflammatory factor expression, showing significant anti-inflammatory activity in LPS-induced macrophage models (Malik et al., 2023). Details are shown in the Table 1.

Accumulating evidence from multiple randomized controlled trials (RCTs) has established the clinical utility of plant extracts as adjunctive therapies for asthma, demonstrating clear potential in alleviating symptoms, optimizing pulmonary function, and modulating inflammatory biomarkers. For instance, a double-blind intervention study involving 36 patients with mild-to-moderate asthma showed that oral administration of *Zataria multiflora* Boiss. [Lamiaceae; Zatariae multiflorae herba] ethanol extract (containing 7.7 mg/100 mL carvacrol and 55.4 mg/100 mL thymol) significantly reduced daytime and exercise-induced wheezing, improved forced vital capacity (FVC), peak expiratory flow (PEF), and maximal mid-expiratory flow (MMEF), while decreasing total leukocyte and eosinophil counts, high-sensitivity C-reactive protein (hs-CRP) levels, and increasing IL-10 expression in peripheral blood mononuclear cell (PBMC) supernatants (Alavinezhad et al., 2022). Similarly, treatment with *Crocus sativus* L. [Iridaceae; Croci stigma] at 100 mg/day for 8 weeks in 80 patients with mild-to-moderate allergic asthma significantly lowered hs-CRP and anti-HSP70 antibody levels and improved FEV1, FVC, and forced expiratory flow at 25%–75% of vital capacity (FEF25–75) (Hosseini et al., 2018). Clinical data on Nigella sativa L. [Ranunculaceae; Nigellae sativae semen] preparations further support these findings: black seed oil containing 0.7% thymoquinone administered at 500 mg twice daily for 4 weeks significantly improved Asthma Control Test (ACT) scores and reduced peripheral eosinophil proportions in 80 patients with uncontrolled asthma; additionally, its powdered formulation enhanced FEF25%–75%, reduced PEF variability and fractional exhaled nitric oxide (FeNO) levels, and elevated serum IFN-γ in 76 patients with partially controlled asthma over 12 weeks (Koshak et al., 2017; Salem et al., 2017). The adjunctive efficacy of plant extracts is further corroborated by studies on *Magnolia biondii* Pamp. [Magnoliaceae; Magnoliae flos] extract (NDC-052), which, when added to inhaled corticosteroids in 148 patients with mild-to-moderate asthma for 8 weeks, significantly improved morning and evening PEF and overall symptom scores (Park et al., 2012). Moreover, *Hedera helix* L. [Araliaceae; Hedera helicis folium] dry extract combined with inhaled corticosteroids significantly enhanced forced expiratory flow between 75% and 25% of FVC (MEF75–25), MEF at 25% of FVC (MEF25), and vital capacity (VC) in 30 children with uncontrolled mild asthma after 4 weeks of treatment (Zeil et al., 2014). Collectively, these RCTs underscore the evidence-based value of plant extracts in the long-term management of asthma. Their multi-component and multi-target mechanisms offer important complementary therapeutic options for optimizing existing treatment regimens and improving patient outcomes. Details are shown in the Table 3.

**TABLE 3:** Clinical studies on botanical extracts and phytochemical metabolite for asthma management.

Intervention category	Intervention name	Study population	Doseage	Treatment duration	Outcome measures	References
Botanical extracts	*Zataria multiflora* Boiss. ethanol extract	36 patients with mild-to-moderate asthma	5 or 10 mg/kg/day	2 months	↑FVC; ↑PEF; ↑MMEF ↓Total WBC; ↓Eosinophils; ↓hs-CRP; ↑PBMC IL-10	Alavinezhad et al. (2022)
	*Crocus sativus*L.	80 patients with mild-to-moderate allergic asthma	100 mg/day	8 weeks	↑FEV1; ↑FVC; ↑FEF25-75 ↓hs-CRP; ↓Anti-HSP70 antibody	Hosseini et al. (2018)
	*Nigella sativa*L. oil	80 patients with uncontrolled asthma	500 mg bid	4 weeks	↑ACT score; ↓ Blood eosinophils	Koshak et al. (2017)
	*Nigella sativa*L. powder	76 patients with partly controlled asthma	1 or 2 g/day	12 weeks	↑FEF25%-75%; ↓PEF variability; ↓FeNO ↑Serum IFN-γ	Salem et al. (2017)
	*Magnolia officinalis*Rehder & E.H.Wilson extract (NDC-052)	148 patients with mild-to-moderate asthma (on ICS)	600 mg/day	8 weeks	↑Morning PEF; ↑Night PEF	Park et al. (2012)
	*Hedera helix* L. dried leaf extract	30 uncontrolled mild asthmatic children (on inhaled steroids)	70 mg/day	4 weeks	↑MEF75-25; ↑MEF25; ↑VC	Zeil et al. (2014)
Phytochemical	*Sulforaphane*	45 patients with moderate asthma	100 μM/day	14 days	↓MCh challenge post-FEV1 decline ↓Small airway resistance	Brown et al. (2015)
	*Plant sterol esters*	Asthma patients (subtype not specified)	4 g/day	Not specified	↓Total IgE; ↑Vaccine-induced antibody response	Brüll et al. (2016)
	*Glycine max(L.) Merr.* [Fabaceae] isoflavones	345 patients with poorly controlled asthma	100 mg/day	24 weeks	No significant improvement in FEV1 or lung function; No significant clinical symptom improvement	Smith et al. (2015)
	*Berry polyphenol extract*	28 steroid-naïve asthma patients	1,000 mg/day	4 weeks	FeNO showed no significant decrease (*p* = 0.98)	Power et al. (2017)

## 7 Isolated phytochemicals

In summary, flavonoids, phenolic acids, and polyphenolic compounds are the core active ingredients in plant medicines for alleviating asthma. Their mechanisms mainly cover multiple dimensions such as anti-inflammation, antioxidant, immunomodulation, and anti-remodeling.

### 7.1 Flavonoids

Flavonoids, as plant secondary metabolites, demonstrate significant anti-inflammatory and antioxidant potential in reducing IgE receptor expression, regulating signal transduction, and reducing inflammatory mediator release (Castell et al., 2014; Bondonno et al., 2024).

Most flavonoids exert core antioxidant effects by activating the Nrf2/HO-1 pathway. Myricetin, widely present in berries and tomatoes, not only reduces Th2 cytokines and CCL11, CCL24 eosinophil chemokines but also activates the Nrf2/HO-1 pathway, promoting SOD and GSH production and reducing MDA levels, thereby alleviating oxidative damage (Huang et al., 2024a). In lung tissue, myricetin promotes antioxidant enzyme production, reduces MDA activity, and alleviates oxidative damage via Nrf2/HO-1 pathway activation. Linarin (LIN) enhances SOD, CAT, and GSH activities, reduces MDA, inhibits inflammatory cell infiltration, decreases IL-4/IL-13/TNF-α, lowers IgE, and inhibits histamine release through dual antioxidant and anti-inflammatory mechanisms (Shen et al., 2025). Ding N et al. confirmed that LIN can directly bind ALDH2, stabilize its expression, and then downregulate MAOA, forming an ALDH2/MAOA axis. This axis inhibits mitochondrial fission, promotes mitochondrial fusion, reduces oxidative stress and mtDNA leakage, thereby inhibiting the cGAS-STING inflammatory pathway and ferroptosis (Ding et al., 2025a). Quercetin (QCT), a widely distributed flavonoid in plants, has multiple effects including antioxidant, anti-inflammatory, and immunomodulatory actions. Besides activating Nrf2 to upregulate GCLC and NQO1 expression, recent studies also found it can inhibit ferroptosis via the SIRT1/Nrf2/HO-1 signaling pathway, reducing asthma airway inflammation and iron overload (Rajizadeh et al., 2023; Winnica et al., 2025; Sun et al., 2024). Ding N et al. confirmed in HDM-induced asthma models *in vivo* and *in vitro* that artemetin directly binds ABCG2, upregulates its expression, inhibits RAB7A, reduces DRP1-mediated mitochondrial fission, thereby alleviating oxidative stress (Ding et al., 2025b). Xanthohumol (XN), an isoprenylated flavonoid derived from Humulus lupulusL., has been shown by Chen et al. to reduce the accumulation of ROS while enhancing SOD and GSH activities, thereby mitigating mitochondrial membrane potential loss and double-stranded DNA (dsDNA) release. *In vitro* experiments demonstrated that XN inhibits the assembly of the AIM2 inflammasome and the subsequent secretion of downstream cytokines IL-1β and IL-18. These findings confirm that XN suppresses ROS-mediated oxidative stress, reduces mitochondrial damage and dsDNA leakage, thereby blocking the dsDNA-activated AIM2 inflammasome pathway (Chen et al., 2026). Tectorigenin (Tec) promotes Nrf2 nuclear translocation by inhibiting Keap1, activates downstream antioxidant gene HO-1 expression, clears ROS, and reduces oxidative damage; simultaneously regulates Th1/Th2 balance and inhibits EMT (Jiang et al., 2024).

Numerous studies confirm that flavonoids can extensively target key inflammatory pathways like TLR4/NF-κB and MAPK. Baicalin (BA), the effective active ingredient of the traditional plant medicine Scutellaria baicalensis, significantly alleviates inflammation and collagen deposition in pediatric allergic asthma models by upregulating miR-103 and inhibiting the TLR4/NF-κB axis (Zhai and Wang, 2022). Wogonoside, also from Scutellaria baicalensis, inhibits the NF-κB/STAT6 pathway, reducing Th2 factor release and mucus secretion, thereby alleviating asthma symptoms (Yu et al., 2024c). La Yi et al. demonstrated in OVA-induced allergic asthma mouse models that formononetin (FMT) regulates Th2/Th17 immune responses and reduces oxidative stress through multiple pathways, including inhibiting the NF-κB/JNK signaling pathway and activating Nrf2/HO-1 (Yi et al., 2020). Breviscapine (Bre) specifically inhibits the TLR4/MyD88/TRAF6 pathway, reducing epithelial cell inflammation and mucus hypersecretion, an effect dependent on TLR4 expression levels. Naringenin and neohesperidin improve allergic asthma symptoms by reducing MDA, increasing GSH, and inhibiting IL-4/IL-13, with efficacy comparable to prednisolone (Cheng et al., 2025; Jasemi et al., 2022).

For airway remodeling, multiple flavonoid components show potential in inhibiting smooth muscle proliferation and fibrosis. Icariin (ICA) and its derivative icaritin (IC) not only alleviate inflammation via the Nrf2/NF-κB axis, but IC also upregulates PD-1 expression, reducing inflammatory cell infiltration and apoptosis in RSV-infected asthma mice (Wang et al., 2026; Fu and Wang, 2025). Epimedin C negatively regulates the non-canonical NF-κB p52/RelB pathway and inhibits ERK1/2 and p38 MAPK activation, regulating the balance between Th9 and Treg cells, thereby alleviating asthma pathology. Its shows advantages in regulating Treg cell function (Huang et al., 2020). Wang SY et al. revealed that luteolin inhibits excessive autophagy through dual pathways: activating PI3K/Akt/mTOR signaling and inhibiting the Beclin-1-PI3KC3 complex, thereby reducing goblet cell hyperplasia and collagen deposition (Wang et al., 2021b). Tectorigenin also inhibits lung fibrosis by downregulating the TGF-β1/Smad pathway and reducing hydroxyproline deposition (Wang et al., 2020). Furthermore, Huang et al. demonstrated in gene knockout mice and cell co-culture models that myricetin activates Sirt1, inhibits the JNK/Smad3 pathway, reduces macrophage inflammatory factor release, thereby blocking fibroblast activation and collagen deposition (Huang et al., 2024b).

Notably, some flavonoids also play unique roles in immunomodulation and specific targeting. Apigenin regulates the Bcl-2/Bax/Caspase-3 axis, reduces EC apoptosis, and improves mitochondrial morphology, effectively alleviating chronic obesity-associated asthma’s non-eosinophilic inflammation, oxidative stress, and airway cell apoptosis (Yu et al., 2023). Sophoraflavanone G (SG), an active ingredient from Sophora flavescens, indirectly alleviates airway inflammation and remodeling by inhibiting Th2 activation and enhancing antioxidant enzyme activity (Wang et al., 2022). Calcaratarin D (CalD) is a novel anti-inflammatory natural compound that covalently binds and targets JAK1/STAT6 and FoxO1/IRF4 pathways, blocks alveolar macrophage M2 polarization, and restores the Nrf2 antioxidant pathway (Liao et al., 2023). Yang et al. demonstrated that in a murine obese asthma model, involucrasin B (IB) exerted comparable efficacy to DEX in alleviating airway hyperresponsiveness (AHR). Notably, IB concurrently suppressed pulmonary pathogenic Th17 and ILC3 expansion, blocked IL-17A-driven neutrophilic infiltration, and ameliorated obesity-associated dyslipidemia. In contrast, DEX failed to restrain Th17 proliferation, exerted only modest inhibitory effects on neutrophil extracellular trap (NET) formation, and lacked regulatory capacity against dyslipidemia. Collectively, these findings highlight the unique therapeutic potential of IB for corticosteroid-resistant obese asthma, addressing key limitations of current glucocorticoid regimens (Yang et al., 2024).

### 7.2 Triterpenoid saponins

Triterpenoid saponins, widely found in traditional medicinal plants, exhibit multi-target pharmacological activity in asthma treatment, primarily exerting effects by regulating oxidative stress, autophagy, and immune imbalance.

Cycloastragenol (CAG) from Astragaluswas shown to alleviate OVA-induced airway inflammation, mucus hypersecretion, and AHR in mice by downregulating autophagy key proteins like LC3B, p62, and Beclin-1, inhibiting excessive autophagy (Zhu et al., 2021). Another compound, Astragaloside IV, exerts protective effects through a dual mechanism: on one hand, inhibiting NF-κB and MAPK signaling pathways, reducing IL-6, IL-8, CCL5, MCP-1, CCL11/24/26, and ICAM-1 expression, blocking monocyte adhesion; on the other hand, activating the HO-1/Nrf2 antioxidant pathway, reducing intracellular ROS levels, and alleviating oxidative damage (Hsieh et al., 2022). Ginsenoside Rg3, extracted from Panax ginseng, also enhances GSH and SOD activities in lung tissue, reduces MDA levels, activates the Nrf2/HO-1 pathway, and directly inhibits NF-κB signaling to reduce ICAM-1-mediated immune cell adhesion, thereby maintaining airway barrier integrity (Huang et al., 2021).

Betulin reduces tTG/TGF-β1/MMP-9 expression, regulates Th1/Th2 imbalance, achieving significant anti-inflammatory and anti-remodeling effects, alleviating OVA-induced airway inflammation and AHR (Kamaraj et al., 2021). Platycoside E (PE) from Platycodon grandiflorum reduces TNF-α, IL-6, and other pro-inflammatory factor release by inhibiting the IL-13/Th2 signaling pathway while regulating redox balance, alleviating airway inflammation and AHR.Mechanistic studies show that high-fat diet (HFD) exacerbates asthma by enhancing Th17 differentiation and adipose tissue inflammation, while PE may antagonize the worsening effect of obesity on asthma phenotype through direct anti-inflammatory and metabolic regulatory effects (Xu et al., 2023).

### 7.3 Polyphenols

Polyphenolic compounds are highly regarded in asthma treatment due to their significant antioxidant and anti-inflammatory properties. They mainly exert multi-target efficacy by regulating ferroptosis, the gut-lung axis, and epithelial barriers.

Curcumin inhibits HDAC8, NF-κB, and MAPK pathways, regulates Th2 cytokine expression, effectively alleviating oxidative stress and airway remodeling (Islam and Singh, 2023). In models induced by the environmental toxin dibutyl phthalate (DBP), DBP exacerbates asthma inflammation and airway remodeling by disrupting iron homeostasis and inhibiting the Nrf2 pathway, promoting ferroptosis. Intranasal administration of N-acetylcysteine (NAC) or curcumin can activate the Nrf2 pathway, upregulate antioxidant genes like GPx4 and SLC7A11, inhibit ferritinophagy, reduce lipid peroxidation and iron accumulation, thereby significantly alleviating airway inflammation and collagen deposition (Singh et al., 2026). Rosmarinic acid (RosA) demonstrates unique immunomodulatory potential. It not only regulates gut microbiota structure, increases the abundance of short-chain fatty acid (SCFA)-producing bacteria, enhances gut barrier function, but also inhibits 5-hydroxytryptamine synthesis to reduce bronchoconstriction reactions and alleviates lung inflammation by inhibiting the TLR4-NF-κB pathway (Guo et al., 2024). In OVA models, RosA works through dual mechanisms: in immunomodulation, it reduces IgE, IL-4, IL-17A levels, increases IFN-γ to restore Th1/Th2 balance; in antioxidant aspects, it reduces NO2 and MDA levels, increases sulfhydryl (SH), SOD, and CAT activities. Vahideh Abbasnia et al. further confirmed that lemon balm extract and its RosA content significantly improve inflammation, smooth muscle hypertrophy, and mucus secretion in asthma models by regulating leukocyte subset balance, and due to its good tolerability, is considered a promising candidate for adjunctive therapy (Abbasnia et al., 2024). Resveratrol (RES) exerts effects through the gut-lung axis and barrier repair. RES can regulate lung microbiota, increasing the abundance of beneficial bacteria like Akkermansia muciniphila; simultaneously regulating gut microecology, increasing butyrate-producing *Bacteroides* abundance and butyrate levels, exerting systemic immunomodulatory effects. Mechanistically, RES upregulates lung epithelial tight junction protein expression, reduces mucin MUC5AC levels, repairs damaged airway epithelial barriers; additionally, it inhibits LPS-induced pro-inflammatory factor release and upregulates the anti-inflammatory factor IL-10, thereby reducing lung inflammation (Alharris et al., 2022).

### 7.4 Terpenoids

Terpenoids demonstrate pleiotropic effects in asthma treatment, with mechanisms covering multiple dimensions such as antioxidant, neuromodulation, and anti-fibrosis.Oleuropein (OLP) exerts protective effects on lung epithelial cells by regulating mitochondrial function, apoptosis pathways, and DNA repair responses. Mechanistic studies show that OLP effectively reduces ROS generation and JC-1 monomer formation, alleviates IL-17A-induced mitochondrial dysfunction, and is proven to activate antioxidant pathways like Nrf2/HO-1, thereby reducing DNA damage and pro-cancer phenotypes (Montalbano et al., 2024). Myrtenol balances the oxidative-antioxidant system by reducing lung tissue MDA levels and restoring SOD activity; it also reduces goblet cell hyperplasia and bronchial smooth muscle thickness, effectively preventing airway remodeling but has no significant effect on epithelial thickness (Bejeshk et al., 2019). Furthermore, studies confirm that myrtenol not only acts on the lungs but also shows unique neuroprotective potential. In OVA-induced asthma models, myrtenol improves asthma-comorbid cognitive impairment and anxiety-like behaviors by inhibiting systemic inflammatory responses, reducing pro-inflammatory factors like TNF-α and IL-6, and regulating GABAergic neurotransmission and blood-brain barrier permeability (Bejeshk et al., 2023). Paeoniflorin (PF), the core active ingredient of the traditional Chinese medicine peony, alleviates airway inflammation by regulating the “oxidative stress-autophagy” axis. PF inhibits OVA-induced mitochondrial dysfunction, reduces ROS generation, thereby blocking excessive autophagy activation and apoptotic signals, ultimately restoring immune homeostasis. Additionally, PF involves regulation of glycolysis metabolic pathways, demonstrating multi-step synergistic mechanisms (Han et al., 2022). Artesunate alleviates airway remodeling by inhibiting p38 MAPK phosphorylation, downregulating pro-fibrotic factors like FIZZ1, reducing collagen deposition, and goblet cell hyperplasia (Zhang et al., 2023a).

### 7.5 Other natural product categories and their anti-asthma mechanisms

Besides the major categories above, various structurally distinct natural products also exert anti-asthma effects through specific targets, mainly covering alkaloids, sterols, coumarins, and polysaccharides.

Sinomenine (Sin) alleviates OVA-induced asthma airway inflammation and remodeling by downregulating the TGF-β1/Smad3 signaling pathway and inhibiting the epithelial-mesenchymal transition (EMT) process. In this pathway, FIZZ1, a key factor produced by epithelial cells, eosinophils, and M2 macrophages, can directly stimulate lung fibroblasts to overexpress type I collagen and α-smooth muscle actin, leading to ECM deposition; sinomenine blocks this pathological chain by inhibiting its upstream signals (He et al., 2021). The polyacetylene falcarindiol (FAD) dose-dependently alleviates airway inflammation, oxidative stress, and epithelial apoptosis by activating the Nrf2 pathway. Mechanistic validation shows that both *in vivo* Nrf2 shRNA knockdown and *in vitro* ML385 inhibition significantly weaken FAD’s protective effects, confirming Nrf2 as the essential mediator of its anti-asthma effect (Jiang et al., 2026). The phenolic acid gentisic acid (GA) directly binds Keap1, releases its inhibition on Nrf2, promotes Nrf2 nuclear translocation and HO-1 expression, thereby reducing oxidative stress and ferroptosis (Abdelmawgood et al., 2025). Additionally, mulberroside A (MuA) extracted from Morus albaL. alleviates airway inflammation and remodeling by regulating Th2 immune response and oxidative stress (Liou et al., 2025).

The sterol stigmasterol directly antagonizes NK1-R, downregulating inflammatory factors like IL-4 and IL-5, alleviating airway hyperresponsiveness and mucus secretion (Zhang et al., 2023b). The coumarin auraptene (AU) specifically inhibits NF-κB and MAPK pathways, significantly reducing macrophage inflammatory mediators induced by LTA. Notably, AU does not affect HO-1, CAT, or GSH levels while exerting anti-inflammatory effects, suggesting it acts independently of antioxidant mechanisms, distinguishing it from most natural products (Hsia et al., 2021). The lignan arctiin inhibits p38 MAPK phosphorylation and downstream NF-κB nuclear translocation, reduces Th2 cytokine release, and enhances SOD/GSH antioxidant capacity, indirectly inhibiting inflammatory pathway activation (Yuan and Sun, 2024). Schisandrin A inhibits NF-κB, improves E-cadherin/β-catenin membrane localization, regulates Th1/Th2 balance, and inhibits α-SMA expression and collagen deposition (Qiu et al., 2023). The phenylpropanoid cinnamaldehyde (Cinn) alleviates airway inflammation by inhibiting NF-κB p65 nuclear translocation and regulating redox balance (Nour et al., 2026). Isorhapontigenin, a natural stilbene compound extracted from Gnetum cleistostachyum, regulates Th1/Th2 balance, inhibits eosinophil infiltration by inhibiting NF-κB/COX-2 pathway and activating Nrf2/HO-1 pathway (Huang et al., 2026).

Zhang et al. discovered using obese-asthma mouse models that plumbagin (PLB) not only reduces obesity phenotype but also alleviates asthma symptoms by inhibiting inflammatory cell infiltration, lowering pro-inflammatory factor levels, alleviating oxidative stress, and activating the AMPK phosphorylation pathway (Zhang et al., 2023c). Salidroside alleviates AHR, Th2 inflammation, and airway remodeling by regulating pyrimidine metabolism, steroid hormone synthesis, and TCA cycle (Wang et al., 2023). Cordycepin (Cor) effectively inhibits OVA-induced asthma airway inflammation and remodeling by upregulating A2AAR expression, downregulating TGF-β1, and inhibiting the p38 MAPK pathway, and has a synergistic effect with budesonide (Fei et al., 2017). Cordyceps militarispolysaccharide (CMP) significantly alleviates oxidative stress, inflammatory responses, and tissue damage by activating the Nrf2/HO-1 pathway, inhibiting the NF-κB pathway, and regulating gut microbiota homeostasis (Song et al., 2023). Furthermore, Cordyceps militarispolysaccharide is also proven to reduce inflammatory cell infiltration and mucus secretion by downregulating TGF-β1 and p-Smad2/3 expression, thereby alleviating airway remodeling and AHR (Zheng et al., 2020). Ephedrae Botanical drug apoly saccharides exert multi-pathway anti-asthmatic effects by inhibiting inflammatory factor release, reducing oxidative stress, regulating DC function, and rebuilding Treg/Th17 balance (Zhang et al., 2022). Details are shown in the Table 2.

### 7.6 Clinical evidence for plant-derived interventions in asthma

Clinical investigations into phytochemicals have yielded pivotal insights, albeit with marked heterogeneity in therapeutic outcomes across different agents. Sulforaphane administration (100 μmol/day) for 14 days attenuated methacholine (MCh)-induced declines in FEV_1_ by 21% in 60% of participants and reduced small airway resistance in a cohort of 45 patients with moderate asthma (Brown et al., 2015). Furthermore, a randomized RCT involving 58 participants demonstrated that phytosterol esters, while not directly enhancing lung function parameters, modulated Th1/Th2 immune homeostasis, resulting in significant reductions in serum total IgE, total cholesterol, and low-density lipoprotein cholesterol (LDL-C), alongside augmented vaccine-specific antibody responses. These immunomodulatory and metabolic effects suggest a promising therapeutic niche for phytosterol esters as adjunctive treatments, particularly in obesity-associated asthma phenotypes (Brüll et al., 2016). Conversely, the clinical benefits of certain bioactive constituents remain unsubstantiated. In a large-scale RCT involving 345 patients with poorly controlled asthma, supplementation with soy isoflavones (100 mg/day) for 24 weeks failed to yield significant improvements in FEV_1_ or clinical symptomatology (Smith et al., 2015). Similarly, a 4-week intervention with berry polyphenol extract (1,000 mg/day) did not reduce FeNO levels in 28 steroid-naïve asthma patients (p = 0.98) (Power et al., 2017). Such null findings possess considerable academic value. Constrained by ethical oversight, financial investment, and technical feasibility, clinical endpoints predominantly focus on macroscopic clinical manifestations, often precluding comprehensive assessment of molecular signal transduction and multi-omics alterations. Consequently, negative results serve to delineate the therapeutic boundaries of a specific compound within a defined patient phenotype but do not negate its potential regulatory effects in alternative asthma endotypes or molecular pathways. These outcomes provide essential direction for subsequent precision medicine and stratified clinical translation research. Details are shown in the Table 3.

## 8 Discussion

The pathogenesis of asthma represents a self-amplifying, closed-loop cascade reaction mediated by the epithelial barrier–immune response axis. Airway epithelial cells serve not only as the initiation point of this cascade but also as the central target for inflammation resolution and barrier restoration. Their consistent involvement across all endotypes has established them as a focal point in asthma research. However, while current first-line clinical therapies suppress immune activity and modulate signaling pathways, they lack specific mechanisms to correct oxidative and antioxidant imbalances or directly restore epithelial barrier integrity. This leaves a critical therapeutic gap, particularly for patients with refractory asthma or Type 2-low endotypes.

Globally, botanical drugs occupy a central role in traditional medical systems, offering a vast repository of structurally diverse metabolites. Evidence synthesized in this review demonstrates that specific plant extracts and their bioactive constituents achieve therapeutic efficacy comparable to glucocorticoids in preclinical models. Crucially, these agents possess unique multidimensional intervention advantages, concurrently attenuating oxidative stress, suppressing mucus hypersecretion, and inhibiting airway remodeling alongside their anti-inflammatory effects. This capacity enables botanical drugs to broaden the therapeutic scope of conventional pharmacotherapies, positioning them as ideal adjunctive treatments for asthma. For mild-to-moderate cases or populations intolerant to corticosteroids, they also hold considerable promise as alternative therapies. Medicinal plants enriched with multi-component synergistic systems capable of simultaneously modulating upstream alarmin release and downstream structural remodeling pathways. This regulatory paradigm suggests that botanical interventions may address mechanistic blind spots inherent to single-target synthetic drugs.

Despite these promising prospects, significant bottlenecks impede clinical translation. First, the existing evidence base remains heavily skewed toward superficial mechanistic explorations *in vitro* and in murine models, lacking depth in pharmacokinetic relationships, long-term toxicological characterization, and especially systematic evaluations of efficacy in hμMan subjects. Furthermore, overcoming pharmacokinetic barriers is essential. The poor systemic bioavailability of many phytochemicals such as curcumin necessitates advanced formulation strategies, including nanoemulsions and liposomes, to enhance pulmonary deposition and local target engagement. Crucially, clinical evaluations must extend beyond subjective symptom scores to include objective hμMan pharmacodynamic biomarkers. Metrics such as fractional exhaled nitric oxide for T2 inflammation, urinary 8-isoprostane for oxidative stress, and peripheral blood transcriptomic signatures can provide concrete evidence of target engagement, thereby bridging the gap between preclinical cellular mechanisms and clinical efficacy. Second, while traditional medicine provides invaluable guidance through syndrome-differentiated prescriptions of complex herbal decoctions, modern research overwhelmingly focuses on isolated extracts or single purified compounds. This disparity creates a conspicuous disconnect, as high-quality clinical data supporting the use of standardized extracts or pure compounds in asthma patients remain notably scarce. A prerequisite for addressing this translational chasm is the rigorous standardization of botanical preparations. Unlike single-molecule synthetic drugs, the chemical complexity of plant extracts introduces significant challenges, including batch-to-batch variability arising from genetic, geographical, and seasonal factors, as well as inconsistencies in extraction methodologies. Future investigations must prioritize the establishment of validated chromatographic markers and chemical fingerprints, adhere to stringent pharmacopeial monographs, and maintain voucher specimens for authentication. Addressing contamination and adulteration risks is equally critical to ensure reproducible pharmacological effects and regulatory compliance. Third, the inherent complexity of botanicals presents formidable challenges for quality control. Batch-to-batch variability, geographic variation in cultivation affecting phytochemical composition, and inconsistent extraction methodologies contribute to significant fluctuations in bioactive compound concentrations. These factors not only undermine inter-study reproducibility but also constitute major obstacles to regulatory approval and standardized clinical application. Moreover, safety evaluations warrant particular attention. While natural origin is often erroneously equated with safety, botanical drugs carry risks of hepatotoxicity, allergenicity, and immunosuppression. A critical yet often overlooked aspect is herb-drug interactions, especially in asthma patients concurrently using inhaled corticosteroids, long-acting beta2-agonists, leukotriene receptor antagonists, or biologics. Systematic preclinical and clinical assessments of these interactions are mandatory. Additionally, robust safety data regarding use during pregnancy and lactation—periods requiring meticulous asthma control—must be established to define clear risk-benefit profiles. Furthermore, a notable methodological limitation observed across the literature is the prevalent reporting bias favoring positive outcomes, wherein negative results and comprehensive safety evaluations are frequently omitted, potentially introducing bias into this review.

Given the substantial heterogeneity among the included studies—particularly regarding experimental models, dosing regimens, and outcome measures—along with the unavailability of individual participant data for pooled analysis, this review adopts a narrative approach rather than a quantitative meta-analysis. Our primary aim is to synthesize current evidence regarding the mechanistic alignment between botanical agents and asthma endotypes, thereby providing a conceptual framework for future translational research. Nevertheless, the aforementioned bottlenecks underscore the need for cautious interpretation of the current evidence base.

To bridge the chasm between traditional knowledge and modern evidence-based medicine, future investigations must advance across multiple dimensions. Mechanistic studies should transcend simplistic pathway mapping to elucidate the precise molecular interactions between botanical constituents, epithelial barriers, and immune checkpoints. Concurrently, rigorous chemometric analyses and stability assessments must be implemented to ensure batch-to-batch consistency of extracts. Parallel to these efforts, translational research should prioritize well-characterized, standardized botanical preparations, spearheading the design and execution of high-standard, multicenter randomized controlled trials—with particular emphasis on efficacy evaluation in specific patient subpopulations, such as those with corticosteroid-resistant asthma or overlapping inflammatory endotypes. Critically, clinical trial design must evolve to align with asthma heterogeneity. Future RCTs should move away from broad inclusion criteria and adopt endotype-stratified approaches, segregating patients based on T2-high biomarkers such as fractional exhaled nitric oxide and eosinophils versus neutrophilic or mixed inflammation. Enriching cohorts with steroid-refractory patients represents a high-impact strategy for demonstrating adjunctive benefits. Trials must employ validated endpoints—including the Asthma Exacerbation Rate, Asthma Control Questionnaire and Asthma Control Test scores, forced expiratory volμMe in one second, and fractional exhaled nitric oxide—while respecting minimal clinically important differences. Moreover, extending trial durations beyond acute rescue effects is necessary to evaluate impacts on exacerbation frequency and potential surrogate markers of airway remodeling. Exploring advanced drug delivery systems, including nanoemulsions and liposomes, remains imperative to enhance the bioavailability of poorly water-soluble phytochemicals and achieve targeted pulmonary deposition, thereby providing robust technological support for the precise clinical application of botanical drugs. In conclusion, botanical drugs harbor immense potential to transform asthma management by intervening in pathological facets currently neglected by conventional therapies. Overcoming barriers related to mechanistic depth, standardization, and clinical validation will be essential to fully realizing their value as safe, effective, and sustainable complementary or alternative therapeutic options in the global fight against asthma.

## 9 Conclusion

In conclusion, this review systematically elucidates the unique advantages of plant-derived natural products in the multidimensional intervention of asthma. Beyond serving as a crucial molecular bridge connecting traditional empirical knowledge with modern pharmacological science, purified natural products offer a viable solution to the longstanding bottlenecks of quality control and standardization that challenge complex traditional formulations. Distinct from the single-target limitations of synthetic drugs, these bioactive constituents exhibit sophisticated “multi-target, multi-pathway” synergism. As detailed in our mechanistic analysis, they concurrently antagonize epithelial-derived alarmins, suppress oxidative stress, rebalance immune responses, and inhibit structural airway remodeling. This poly pharmacological profile aligns intrinsically with the pathophysiological complexity of asthma, rendering them exceptionally suited to address disease heterogeneity. Consequently, botanical agents represent not merely alternative therapies, but indispensable adjunctive options poised to fill the therapeutic void in refractory asthma and Type 2-low endotypes. Future translational success, however, hinges on rigorous endotype-stratified clinical trials and the integration of quantitative biomarkers to validate their efficacy beyond conventional metrics.
